# Factors associated with commercial sexual activities among students engaging in casual heterosexual behaviors: a cross-sectional study in Zhejiang Province

**DOI:** 10.3389/fpubh.2025.1665110

**Published:** 2025-11-18

**Authors:** Zhongrong Yang, Qiaoqin Ma, Weiyong Chen, Wanjun Chen, Xin Zhou, Tingting Jiang, Hui Wang, Yaping Yao

**Affiliations:** 1 Department of HIV/TB Control and Prevention, Huzhou Center for Disease Control and Prevention, Huzhou, Zhejiang, China; 2 Department of HIV/STD Control and Prevention, Zhejiang Provincial Center for Disease Control and Prevention, Hangzhou, Zhejiang, China; 3 Zhejiang Key Lab of Vaccine, Infectious Disease Prevention and Control, Hangzhou, Zhejiang, China

**Keywords:** commercial sexual behavior, factors, heterosexual behavior, HIV/AIDS, students

## Abstract

**Objective:**

This study aimed to investigate the occurrence of and factors associated with commercial sexual activities among college students who engaged in casual heterosexual behaviors in the past year.

**Methods:**

Using an independent self-designed online survey questionnaire, information on demographic characteristics, attitudes towards sex, HIV prevention and control knowledge, and intervention acceptance was collected. Logistic regression analysis was used to determine factors associated with commercial sexual behavior among college students who had engaged in casual heterosexual behavior in the past year.

**Results:**

In total, 42,380 students were surveyed and 440 incomplete questionnaires were excluded, resulting in 41,940 valid responses. Among them, 2,581 college students reported involvement in heterosexual activities in the previous year, representing 6.15% of the total student population. Specifically, 425 college students reported engaging in casual heterosexual behavior in the previous year, accounting for 16.5% of students who engaged in heterosexual activities. Of these, 74 (17.4%) students had engaged in commercial sex (average age, 19.99 ± 1.22 years). Multivariate logistic regression analysis indicated that accepting commercial sex [adjusted odds ratio (aOR) 7.33, 95% confidence interval (CI) 3.24–16.58], opposite-sex partners being non-students (aOR2.48, 95% CI 1.24–4.99), recent anal intercourse (aOR3.11, 95% CI 1.33–7.28), seeking casual partners on the Internet rather than offline (aOR2.33, 95% CI 1.19–4.56), perceived risk of HIV infection (aOR2.93, 95% CI 1.13–7.59), and consistent condom use during casual sex (aOR0.27, 95% CI0.12–0.65) or sometimes/often use (aOR0.26, 95% CI0.11–0.64) compared with never using a condom were independent factors associated with the occurrence of commercial sex among college students who had engaged in casual heterosexual behaviors in the past year.

**Conclusion:**

Commercial sexual activity among college students who engaged in casual heterosexual behavior was relatively common in Zhejiang Province, and was characterized with a high degree of openness towards sex, a low perception of HIV risk, low condom usage, and knowledge-practice separation. Strengthening HIV risk warnings and sex education tailored to this group is recommended, to promote the integration of knowledge and action, increase condom usage rates, and reduce the occurrence of unsafe sexual behavior.

## 1 Introduction

Acquired immunodeficiency syndrome (AIDS) is a systemic disease owing to human immunodeficiency virus (HIV) infection (1) that can induce varying degrees of immune deficiency in individuals. Untreated patients are vulnerable to severe infections and malignancies, leading to fatal outcomes (2, 3). The current global HIV epidemic remains severe, with 85.6 million infections and 40.4 million AIDS-related deaths globally, particularly in developing nations, where AIDS persists as a fatal disease (4, 5). As of 2024, China has reported over 1.3 million people infected with HIV-1 and over 470,000 deaths from the virus (6). Despite the implementation of diverse governmental measures worldwide to combat HIV/AIDS, including educational initiatives, free HIV testing, and antiretroviral therapies, various challenges remain such as societal stigmatization towards HIV-infected populations and sluggish advancements in AIDS vaccine development (3, 7, 8).

The escalating incidence of HIV/AIDS among Chinese students in recent years has gained significant attention, with this group being increasingly focused on in AIDS prevention and control endeavors. Sexual transmission stands out as a primary factor driving the mounting HIV/AIDS rates among Chinese students (9). Greater societal openness and evolving perceptions towards sexuality have also fostered inappropriate sexual conduct and a blurred understanding of sexual notions within teenage and university student populations (10). Primarily stemming from a deficiency in accurate sexual health education, many students lack awareness regarding sexually transmitted disease risks, possess a limited understanding of HIV/AIDS transmission channels and prevention strategies, and exhibit a dearth of self-protection consciousness, amplifying their vulnerability to HIV infections (11, 12). To effectively counter the swiftly spreading HIV/AIDS epidemic among Chinese students, it is imperative to institute a robust framework for AIDS prevention and control within educational institutions. This entails the implementation of routine health assessments, counseling services, and comprehensive health protection measures to halt the proliferation of HIV/AIDS within the student community (13).

Heterosexual transmission has emerged as the predominant mode of HIV dissemination, responsible for >50% of cases originating from both commercial and noncommercial heterosexual interactions (14). Managing heterosexual transmission poses a formidable challenge for prevention and control. Commercial heterosexual engagement frequently entails a range of risk factors, including improper condom use and frequent sexual encounters, which increase the risk of HIV transmission. Moreover, noncommercial heterosexual activities carry inherent transmission risks, particularly in contexts characterized with limited HIV awareness and prevention measures, where unsafe practices between sexual partners can precipitate HIV transmission. In China, commercial sexual activities are strictly prohibited, with certain social mores and legal frameworks regarding sexual activity firmly entrenched. Therefore, limited attention has been paid to commercial heterosexual activities within the college student demographic, potentially resulting in underestimation of this phenomenon and concomitant exposure to associated risks and hazards (15).

College students, as a cohort of young individuals, find themselves in a critical stage of growth and development, and encounter myriad pressures and temptations emanating from society, family, and peers. Consequently, some students engage in commercial sexual activities driven by economic or other factors. These activities have significant health and social implications, including the risk of contracting sexually transmitted diseases, worsening mental health issues, and blurring moral and ethical boundaries. Given that college students are not yet physically and mentally mature, they may be particularly susceptible to the effects and risks of such activities. As these students transition into post-college life and establish families, understanding the characteristics of commercial sexual activities among those engaged in casual opposite-sex encounters becomes imperative. This study aimed to investigate the features and factors associated with commercial sexual activities among heterosexual college students based on a cross-sectional survey involving participants from 15 universities in Zhejiang Province, offering a scientific foundation for devising appropriate preventive strategies.

## 2 Materials and methods

### 2.1 Study design

This study implemented a cross-sectional survey approach to investigate students from 15 universities located in 11 cities across Zhejiang Province between November and December 2020. To determine the sample size, the following calculation method was applied: utilizing data from behavioral monitoring obtained in repeated surveys, we derived the formula N = 400 × Q / P. Here, P represents the estimated proportion of the relevant behavior occurring during the survey; specifically, it is based on the prevalence rate of sexual behavior among college students which was estimated at 15% (*p* = 0.15) (16), Q = 1−P. Given these parameters, the study necessitates a minimum of 2,267 participants recruited via the internet. The universities were selected based on recommendations from local disease control centers, with five universities situated in Hangzhou and one in each of the other 10 cities. The survey employed stratified cluster sampling. Initially, three departments were randomly selected from each university to ensure a minimum of 800 students in each department. Subsequently, classes were randomly selected within each grade level, with a minimum of 200 students per grade for the four-year program and at least 267 students per grade for the three-year program, encompassing all students in the chosen classes.

### 2.2 Participants

**Figure 1:**
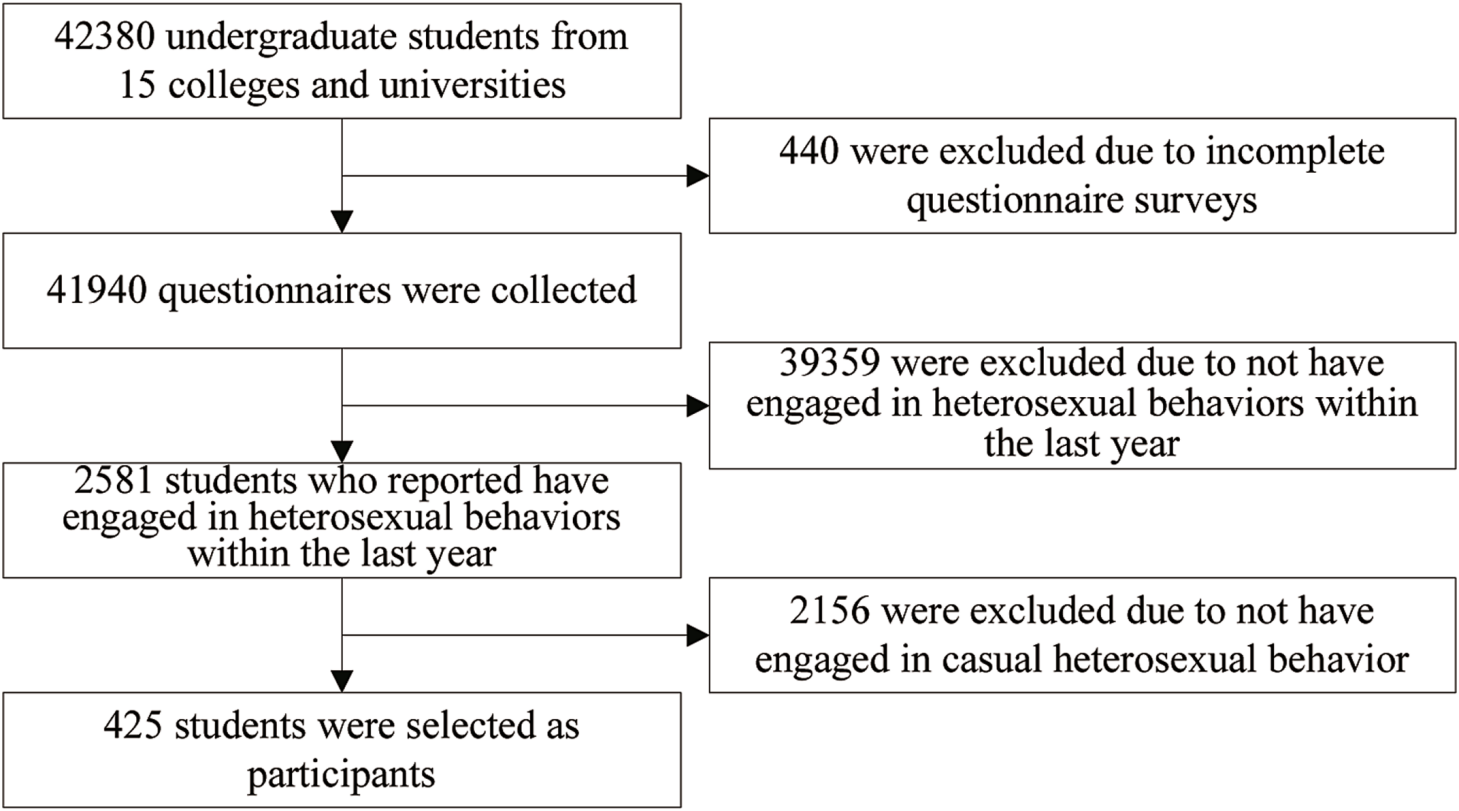
The flowchart for the inclusion and exclusion process.

The participants were college students who reported engaging in casual heterosexual encounters during the previous year. The inclusion criteria for the participants are students from the selected class who are willing to participate in the questionnaire survey with informed consent. The exclusion criteria for participants are students from non sampled classes or those who refuse to participate in the questionnaire survey with informed consent. In total, 42,380 students were surveyed and 440 incomplete questionnaires were excluded, resulting in 41,940 valid responses. Among them, 2,581 college students reported involvement in heterosexual activities in the previous year, representing 6.15% of the total student population. Specifically, 425 college students reported engaging in casual heterosexual encounters in the previous year, accounting for 16.5% of students who engaged in heterosexual activities. Figure 1 illustrates a flowchart detailing the participant inclusion and exclusion processes.

### 2.3 Ethical statement

This study was approved by the Ethics Committee of the Zhejiang Provincial Center for Disease Control and Prevention (CDC) (approval number 2018–036). All the participants provided their written informed consent.

### 2.4 Survey content

Based on a review of domestic and international literature (17, 18), a survey questionnaire was developed through team discussions and pre-surveys were conducted among college students (16). The questionnaire included general demographic characteristics, knowledge of HIV prevention and control, attitudes towards sexuality, intervention and testing situations, risk perceptions, and sexual behavioral characteristics. On-campus students completed an online electronic survey questionnaire (Wenjuanxing) organized by teachers; off-campus students were sent a link to the survey questionnaire and instructed to complete it independently, according to the requirements at the beginning of the questionnaire.

Referring to the study by Hanna et al. (18), a scale for measuring the self-efficacy in condom use was developed, which consisted of three main questions: whether the participant is confident in discussing condom use with a sexual partner before engaging in sexual activity, whether they feel confident in refusing sex or not engaging in sexual activity if the partner does not have a condom, and whether they feel confident in preparing a condom before engaging in sexual activity. Each question had five response options: “very confident,” “quite confident,” “confident,” “not confident,” and “not at all confident,” with corresponding scores of 3, 2, 1, 0, and −1, respectively. The participants were divided according to the total score into three groups: 9 points, 5–8 points, and < 4, a higher score indicates greater self-efficacy (16). The Cronbach’s alpha coefficient for this measurement was 0.823.

### 2.5 Definition of relevant indicators

Casual heterosexual behavior refers to individuals who engage in casual sexual behavior with a heterosexual partner. Commercial sexual behavior refers to individuals who have engaged in commercial sexual activities involving monetary transactions in the past year (such as prostitution). Regarding the type of casual partner, based on the source of casual partners in the past year, participants’ casual partners were categorized as students or non-students (19). According to whether the participants (college students engaging in casual heterosexual behavior) had engaged in commercial sexual behavior in the past year, they were categorized into the following groups: those who engaged in commercial sexual behavior and those who did not.

### 2.6 Statistical analysis

Data analysis was performed using SPSS (version 23.0; SPSS Inc., Chicago, IL, USA) software. Variables including age, sex, household registration, school type, knowledge of HIV prevention and control, attitudes towards sexuality, intervention and testing awareness, risk perception, and sexual behavior characteristics were summarized using means, percentages, or rates. Self-reported involvement in commercial sexual activity in the previous year served as the dependent variable. The independent variables included demographic details, HIV prevention and control knowledge, attitudes towards sexuality, risk perception, intervention awareness, and sexual behavior characteristics. Univariate logistic regression analysis was used to determine the factors associated with college students’ engagement in casual heterosexual behavior and commercial sexual activity in the preceding year. Variables with a significance level of *p* < 0.20 in the univariate analysis were incorporated as independent variables in the multivariate logistic regression model, with statistical significance set at *p* ≤ 0.05.

## 3 Results

### 3.1 Participants’ general demographic characteristics

In total, 425 college students who reported engaging in casual heterosexual behavior in the previous 12 months were included in this study. Among them, 74 (17.4%) students engaged in commercial sexual behavior (average age, 19.99 ± 1.22 years). This group represented 2.9% of college students who reported engaging in heterosexual behavior in the past year. The remaining 351 (82.6%) students (average age, 20.20 ± 1.30 years) did not report engaging in commercial sexual behavior. In the group that engaged in commercial sexual behavior, 27.0% were aged ≤19 years, 93.2% were male, 43.2% were from outside the province, and 68.9% attended regular higher education institutions. The occurrence of commercial sexual behavior was significantly higher among males (20.1%) than among females (6.1%) (*p* < 0.05). No significant differences in age, household registration, or school type were observed between the two groups (Table 1).

**Table 1:** Analysis of factors associated with commercial sexual activity among the participants.

Variables	Commercial sexual activity group (*n* = 74, %)		Non-commercial sexual activity group (*n* = 351, %)		Univariate analysis		Multivariate analysis	
	*n* (%)		*n* (%)		*OR* (95%*CI*)	*p*	a*OR* (95%*CI*)	*p*
Age (yrs)								
≤19	20 (27.0)		128 (36.5)		Ref.		Ref.	
20–21	42 (56.8)		185 (52.7)		1.45 (0.82–2.59)	0.205	1.52 (0.74–3.15)	0.258
≥22	12 (16.2)		38 (10.8)		2.02 (0.91–4.51)	0.086	1.20 (0.41–3.51)	0.747
Sex								
Female	5 (6.8)		77 (21.9)		Ref.		Ref.	
Male	69 (93.2)		274 (78.1)		3.88 (1.51–9.95)	0.005	1.44 (0.43–4.79)	0.55
Registered residence								
Zhejiang Province	42 (56.8)		235 (67.0)		Ref.		Ref.	
Non-Zhejiang Province	32 (43.2)		116 (33.0)		1.54 (0.93–2.57)	0.096	1.46 (0.74–2.88)	0.279
School type								
Higher Vocational and Technical School	23 (31.1)		126 (35.9)		Ref.			
College or universitie	51 (68.9)		225 (64.1)		1.24 (0.73–2.13)	0.431		
Whether the correct use of condoms can reduce the risk of infection and transmission of HIV?								
No/Unknown	23 (31.1)		126 (35.9)		Ref.		Ref.	
Yes	51 (68.9)		225 (64.1)		0.21 (0.10–0.52)	<0.001	0.82 (0.14–4.66)	0.82
Whether AIDS counseling and testing should be actively sought after high-risk sexual behaviors								
No/Unknown	10 (13.5)		11 (3.1)		Ref.		Ref.	
Yes	64 (86.5)		340 (96.9)		0.21 (0.08–0.51)	0.001	0.21 (0.03–1.46)	0.212
Have you received any special lectures on AIDS held by the school in the last year								
No	13 (17.6)		94 (26.8)		Ref.		Ref.	
Yes	61 (82.4)		257 (73.2)		1.72 (0.90–3.27)	0.10	1.65 (0.54–5.11)	0.382
Did you learn about HIV/AIDS through school network in the last year								
No	11 (14.9)		66 (18.8)		Ref.			
Yes	63 (85.1)		285 (81.2)		1.33 (0.66–2.66)	0.425		
Have you received any publicity on HIV testing in schools in the past year								
No	15 (20.3)		101 (28.8)		Ref.		Ref.	
Yes	59 (79.7)		250 (71.2)		1.59 (0.86–2.93)	0.138	1.57 (0.50–4.92)	0.437
Have you accepted the AIDS risk self-assessment carried out by the school in the last year								
No	22 (29.7)		172 (49)		Ref.		Ref.	
Yes	52 (70.3)		179 (51)		2.27 (1.32–3.90)	0.003	1.53 (0.71–3.30)	0.275
Do you accept commercial sexual behavior?								
No/Unknown	11 (14.9)		214(61)		Ref.		Ref.	
Yes	63 (85.1)		137 (39)		8.95 (4.55–17.58)	<0.001	7.33 (3.24–16.58)	<0.001
The sources of casual heterosexual partners								
Student	46 (62.2)		267 (76.1)		Ref.		Ref.	
Non-student	28 (37.8)		84 (23.9)		1.94 (1.14–3.29)	0.015	2.48 (1.24–4.99)	0.011
Have you had anal sex in the past year?								
No	46 (62.2)		326 (92.9)		Ref.		Ref.	
Yes	28 (37.8)		25 (7.1)		7.94 (4.26–14.78)	<0.001	3.11 (1.33–7.28)	0.009
Condom usage during casual sexual activity								
Never use	33 (44.6)		26 (7.4)		Ref.		Ref.	
Sometimes use	17 (23)		117 (33.3)		0.11 (0.06–0.24)	<0.001	0.26 (0.11–0.64)	0.003
Every time use	24 (32.4)		208 (59.3)		0.09 (0.05–0.18)	<0.001	0.27 (0.12–0.65)	0.003
The ways to seek casual heterosexual partners?								
Non-internet	38(51.4)		257 (73.2)		Ref.		Ref.	
Internet	36(48.6)		94 (26.8)		2.59 (1.55–4.33)	<0.001	2.33(1.19–4.56)	0.014
Do you think you are at risk of contracting HIV?								
No/Unknown	60 (81.1)		328 (93.4)		Ref.		Ref.	
Yes	14 (18.9)		23 (6.6)		3.33 (1.62–6.83)	0.001	2.93 (1.13–7.59)	0.027
Self efficacy measurement of condom use (scores)								
≤4	19 (25.7)		79 (22.5)		Ref.			
5–8	15 (20.3)		100(28.5)		0.62 (0.30–1.31)	0.210		
9	40 (54.1)		172 (49)		0.97 (0.53–1.78)	0.914		
Have you received HIV voluntary counseling and testing in the last year								
No	60 (81.1)		323 (92)		Ref.		Ref.	
Yes	14 (18.9)		28 (8)		2.62 (1.34–5.41)	0.005	1.93 (0.75–4.93)	0.171
Do you know that the Center for Disease Control and Prevention provides HIV testing services								
No/Unknown	30 (40.5)		121 (34.5)		Ref.			
Yes	44 (59.5)		230 (65.5)		0.77 (0.46–1.29)	0.322		
Condom usage during sexual intercourse with commercial partners (*n* = 74)								
Never use	27 (36.5)		—					
Sometimes use	19 (25.7)		—					
Every time use	28 (37.8)		—					

### 3.2 Univariate analysis of whether participants engaged in commercial sexual activities

The univariate analysis of whether participants engaged in commercial sexual activities showed that among college students who engaged in casual heterosexual behavior, those who had received HIV risk self-assessment conducted by the school in the past year (OR = 2.27) had also engaged in commercial sexual activities (OR = 8.95), had non-student casual sexual partners as opposed to student partners (OR = 1.94), had engaged in anal sex in the past year (OR = 7.94), had found casual partners on the Internet as opposed to offline (OR = 2.59), perceived themselves at risk of HIV infection (OR = 3.33), had received voluntary HIV counseling and testing in the past year (OR = 2.62), and were more likely to engage in commercial sexual activities. The findings indicated awareness that consistent and correct use of condoms can reduce the risk of HIV infection and transmission (OR = 0.21), that those engaging in high-risk behaviors should actively seek HIV counseling and testing (OR = 0.21), that condoms should be used every time during casual sexual encounters (OR = 0.09), and that occasionally/often using condoms (OR = 0.11) compared with never using them were factors indicating that college students were less likely to engage in commercial sexual activities. There was no statistical difference in the occurrence of commercial sexual activities among college students based on variables such as whether they had attended HIV-themed lectures in the past year, accessed HIV information through the school network, received HIV testing promotions from the school, self-efficacy in using condoms, or awareness of HIV testing services provided by the CDC (Table 1). Further analysis of condom usage among 74 college students engaged in commercial sexual activities revealed that 37.8% used condoms every time, 25.7% used them occasionally/often, and 36.5% never used them.

### 3.3 Multivariate logistic regression analysis

Variables (*p* < 0.2) from the univariate analysis were included in the multivariate logistic regression analysis. The analysis results (Table 1) indicated that the proportion of participants who accepted commercial activities engaging in commercial sexual activities increased by 633% [adjusted odds ratio (aOR) 7.33, 95% confidence interval (CI) 3.24–16.58]. Compared with the group with student casual sexual partners, the proportion of participants with non-student casual sexual partners engaging in commercial sexual activities increased by 148% (aOR 2.48, 95% CI 1.24–4.99); the proportion of participants who engaged in anal sex in the past year and commercial sexual activities increased by 211% (aOR 3.11, 95% CI 1.33–7.28); the proportion of participants who found casual sexual partners on the Internet and who engaged in commercial sexual activities increased by 133% (aOR 2.33, 95% CI 1.19–4.56); and the proportion of participants who perceived themselves at risk of HIV infection and engaged in commercial sexual activities increased by 193% (aOR 2.93, 95% CI 1.13–7.59). Compared with the group who never used condoms, the proportion of participants who consistently used condoms or occasionally used condoms during casual sexual encounters had a 73% (aOR 0.27, 95% CI 0.12–0.65) and 74% (aOR 0.26, 95% CI0.11–0.64) lower likelihood of engaging in commercial sexual activities, respectively.

## 4 Discussion

This study presents findings from a cross-sectional survey of college students in Zhejiang Province, China, focusing on commercial sexual behavior patterns and associated factors related to casual sexual encounters among opposite-sex college students in the province. Our findings indicated that, over the previous 12 months, the prevalence of casual sexual behavior among young students in Zhejiang Province was 16.5% (425 of 2,581), while the prevalence of commercial sexual behavior was 2.9% (74 of 2,581). The ratio of commercial to casual sexual behaviors was 17.4% (74 of 425). In contrast, Ajayi and Somefun (20) reported a considerably higher prevalence (23.8%) of commercial sexual activities involving financial transactions among college students. Moreover, this study found no significant gender-based differences in the occurrence of commercial sexual behavior among college students who engaged in casual sexual encounters. Previous studies have indicated a gradual increase in self-reported sexual behaviors and commercial sexual activities among college students over successive years, alongside a rising trend in new HIV infections across various age groups (21). Consequently, the presence of both casual and commercial sexual behaviors among heterosexual college students in Zhejiang Province underscores the substantial risk of HIV transmission as societal attitudes towards sexuality evolve, highlighting the need for enhanced behavioral surveillance. The results of this study are similar to those of a survey conducted on college students in Jiangxi Province, which indicated that those with sexting experiences are more likely to engage in high-risk sexual behaviors among college students (22).

Commercial sexual activity often involves interactions between partners with undisclosed infection status, thereby increasing the risk of HIV transmission (23). There was a striking increase of 633% in the proportion of participants receptive to engaging in commercial sexual practices. This notable surge implies that a subset of college students who are open to commercial sex may exhibit distinct social or psychological traits that make them more susceptible to the allure and influence of such conduct. These tendencies may be linked to personal financial standing, family background, sexual attitudes, and other determinants. Moreover, this outcome underscores the potentially grave repercussions of commercial sexual behaviors on the well-being and societal dynamics of college students, encompassing the risk of contracting HIV and other sexually transmitted diseases, as well as adverse effects in relation to mental health and interpersonal dynamics. Despite an 86.5% rate among college students in considering HIV counseling after engaging in high-risk sexual activities, only 18.9% (14 of 74) had undergone voluntary counseling and testing, mirroring previous findings that emphasize the gap between knowledge and action in college settings, leading to low HIV testing rates (24). Hence, it is imperative to bolster educational endeavors within HIV prevention initiatives to enhance awareness of risk factors, elevate awareness of disease vulnerabilities among students, promote the amalgamation of knowledge and behavioral responses, boost HIV testing rates, and ultimately, foster informed and secure social engagement.

Jiang et al. (25) demonstrated that involvement in sexual activities among commercial sex workers increases the risk of disease transmission. Study findings revealed a 148% increase in the proportion of non-student individuals engaging in commercial sexual encounters with casual partners of the opposite sex. This result likely signifies a heightened participation rate among non-student individuals in commercial sexual practices. Non-student individuals, when compared with the student population, may possess greater autonomy and social liberties, enabling them to autonomously shape their lifestyles and behaviors, including opting for commercial sexual engagements. Moreover, non-students may exhibit increased social acumen and maturity, affording them a better capacity to navigate the risks and obstacles associated with commercial sexual endeavors. Nonetheless, this outcome may also underscore certain potential risks and challenges encountered by non-student individuals involved in commercial sexual activities, such as increased susceptibility to sexually transmitted diseases and aggravated mental health concerns. Consequently, these findings indicate the need for vigilance in relation to involvement of non-student individuals in commercial sexual activities, prompting the implementation of tailored preventive and supportive interventions.

Some studies have suggested that anal sex carries a heightened risk of transmitting infectious diseases, and various factors influence heterosexual individuals engaging in anal sex, notably curiosity, pursuit of sexual pleasure, and considerations of contraception, with those practicing anal sex being more inclined to seek sexual partners among sex workers or HIV-positive populations (26–28). Our study findings indicated a 211% increase in the proportion of individuals engaged in anal sex over the past year and involved in commercial sexual activities compared with non-participants. Individuals involved in anal sex may be drawn to explore diverse and stimulating sexual behaviors, thus being more open to commercial sexual activities for sexual gratification. Nevertheless, this outcome highlights the heightened health risks and challenges faced by individuals practicing anal sex, and underscore the need for comprehensive health education and support. Therefore, HIV education in university settings should prioritize fostering appropriate sexual ethics and attitudes, promoting a healthy online environment, and discouraging unsafe behaviors, such as commercial sexual activities.

In recent years, the prevalence of online dating has surged, leveraging the Internet as a convenient medium for social interaction and communication (29). This trend has significantly affected individual sexual behaviors and partner choices, facilitating easier and more diverse access to sexual partners, thereby heightening concerns regarding HIV transmission (30). Findings from this study indicated a 133% increase in the proportion of individuals seeking casual partners online and engaging in commercial sexual activities compared with their counterparts who did not use online platforms for casual partnering. This outcome underscores how seeking casual partners online may elevate individuals’ exposure to commercial sexual pursuits given the discreet and widespread promotion of such activities on digital platforms. Furthermore, online searches for casual partners may render individuals more vulnerable to negative influences and enticements, thereby fostering a greater propensity towards involvement in commercial sexual practices. Moreover, this behavior is likely associated with more permissive sexual attitudes and diverse preferences, potentially predisposing individuals to embrace commercial sexual encounters. However, this finding highlights the sexual health risks and complexities faced by individuals in the digital age, emphasizing the need for holistic sexual health education and support. Thus, it is crucial to bolster sexual health education for adolescents and young adults in this Internet-driven era and enforce stringent monitoring and oversight of commercial sexual activities on online platforms to safeguard the well-being of students.

Self-assessment of HIV risk has heightened individuals’ awareness of the potential for HIV infection and promoted HIV testing (31). Findings from this study revealed that individuals who perceived themselves as being at risk of HIV infection demonstrated a 193% increase in involvement in commercial sexual activities compared with those who perceived themselves as not at risk. This observation highlights how varying perceptions of HIV risk influence individuals’ sexual behavioral choices. Those acknowledging the risk of HIV infection may focus more on their health status and the safety of their sexual encounters, leading them to lean towards engaging in commercial sexual activities to mitigate the risk of infection. Such individuals might exhibit increased vigilance towards sexually transmitted diseases, including HIV, thereby showing a greater willingness to adopt protective measures, such as condom use or choosing commercial sexual services to ensure sexual safety. However, this outcome also emphasizes the significance of informed awareness regarding health risks and the notable effect of individual risk perceptions on behavioral decisions. The results of this study underscore the importance of enhancing awareness of sexual health risks among individuals, prioritizing sexual safety, and implementing preventive measures to reduce the occurrence and transmission of sexually transmitted diseases.

The use of condoms is widely acknowledged as an effective measure to reduce the risk of acquiring sexually transmitted diseases (32), whereas engaging in unprotected casual sexual encounters can increase the risk of HIV infection (33). A study showed that 54% of the participants believed that using condoms during sexual intercourse could prevent HIV transmission (34). Findings from this study indicated that individuals who consistently or occasionally used condoms during casual sexual encounters demonstrated a 73 and 74% decrease in engaging in commercial sexual activities, respectively, compared with those who never used condoms. This highlights the critical role of condom use in sexual practices and its positive effect on diminishing instances of commercial sexual engagement. Condom users typically prioritize the safety of their sexual activities, making them more inclined to select safe sexual partners and behaviors, thereby lowering the occurrence of commercial sexual transactions. Our research findings also revealed that some individuals exhibited insufficient condom use during sexual encounters, reflecting a disregard for safety standards and augmenting the risk of contracting sexually transmitted diseases. There is a deficit in the transition among university students from condom knowledge to preventive actions, with the complexity of unsafe behaviors further complicating intervention efforts. Additionally, factors such as accessibility to condoms play a crucial role in determining their utilization. Therefore, in HIV-prevention education for university students, there should be a focus on individual accountability for health and establishing a supportive environment that facilitates condom availability, thus promoting condom use as the foremost strategy for safeguarding against disease transmission.

This study had some limitations. First, the investigation relied on self-reported data from the study participants, particularly involving sensitive and private issues in relation to sexual behavior, which may have introduced certain biases. Owing to the respondents’ potential reluctance or inability to provide honest answers to these questions, the survey results may not have been sufficiently accurate or objective. Therefore, it is crucial to handle these data with caution when interpreting the findings and considering potential biases. The study’s university sample was determined through local CDC recommendations rather than using a stratified sampling method that would consider regional distributions of educational resources, potentially introducing selection bias. This study involved a cross-sectional survey conducted at a specific point in time with the sample; therefore, causal relationships among the identified influencing factors could not be established. Other study designs and data analyses are recommended to establish these causal relationships. Nevertheless, we conducted multiple regression analysis to account for confounding variables and to minimize systematic errors attributed to individual factors. Additional multicenter prospective randomized cohort studies are required to verify our study findings.

## 5 Conclusion

Commercial sexual activities are prevalent among college students who engage in casual heterosexual encounters in Zhejiang Province, and are characterized with a high degree of openness towards sex, a low perception of HIV risk, low condom usage, and knowledge-practice separation. Establishing a platform for sexual health education, strengthening HIV risk warnings education and online monitoring tailored to this group is recommended, in order to promote the integration of knowledge and action, increase condom usage rates, thereby lowering the prevalence of sexually transmitted diseases including HIV. This result of this study is limited by cross-sectional design survey and self-reported data, additional multicenter prospective randomized cohort studies are required to verify the study findings.

## Data Availability

The raw data supporting the conclusions of this article will be made available by the authors, without undue reservation.
